# De Morsier Syndrome Associated With Megalocornea: Impact of Hormone Replacement Therapy on the Resolution of Neonatal Cholestasis

**DOI:** 10.7759/cureus.114713

**Published:** 2026-08-18

**Authors:** Mbaihorem Nicolas, Khellouk Houda, Meriem Atrassi, Dalal Bensabbahia

**Affiliations:** 1 Gastroenterology, Ibn Rochd University Hospital Center, Casablanca, MAR; 2 Pediatric Gastroenterology, Abderrahim El Harrouchi Hospital, Ibn Rochd University Hospital, Casablanca, MAR; 3 Pediatric Gastroenterology, Mother and Child Hospital Abderrahim Harouchi, University Hospital Center Ibn Rochd, Casablanca, MAR

**Keywords:** de morsier syndrome, hypopituitarism, megalocornea, neonatal cholestasis, : septo-optic dysplasia

## Abstract

De Morsier syndrome, or septo-optic dysplasia (SOD), is a rare congenital malformation characterized by optic nerve hypoplasia, midline brain abnormalities, and pituitary hormone deficiencies. Neonatal presentation can be misleading, particularly when it manifests as cholestatic jaundice.

We report the clinical, biochemical, and radiological evaluation of a two-month-old male infant admitted for persistent neonatal cholestatic jaundice, progressive weight faltering, and recurrent hypoglycemia. Slit-lamp examination, comprehensive endocrine workup, and brain magnetic resonance imaging (MRI) were performed to establish the diagnosis. Written informed consent was obtained from the patient's parents (or legal guardians) for publication of this case report and any accompanying images.

Physical examination revealed horizontal nystagmus and bilateral megalocornea (diameter > 13 mm). Laboratory investigations showed cholestasis with normal gamma-glutamyl transferase (GGT) levels and recurrent hypoglycemia, which led to the diagnosis of combined anterior pituitary hormone deficiency (growth hormone, thyroid-stimulating hormone, and adrenocorticotropic hormone). Brain MRI confirmed the diagnosis by showing agenesis of the septum pellucidum, bilateral optic nerve hypoplasia, and an ectopic posterior pituitary gland. Targeted hormone replacement therapy with L-thyroxine (10 μg/kg/day) and hydrocortisone (5 mg/kg/day) led to a complete resolution of the cholestasis within three weeks, alongside blood glucose stabilization and weight gain.

Neonatal cholestasis is a classic early manifestation of SOD due to impaired bile acid transporter maturation caused by endocrine deficits. Its association with megalocornea highlights the variability of embryonic anterior segment anomalies. Early diagnosis is vital to initiate prompt hormone therapy, ensure hepatic recovery, and optimize neurodevelopmental outcomes.

## Introduction

The term septo-optic dysplasia (SOD) was coined by Hoyt et al. in 1970 when they established the link between optic nerve hypoplasia, midline brain abnormalities, and pituitary dwarfism [1]. This built upon the work of the French-Swiss neurologist Georges de Morsier, who in 1956 had first described the association between an absent septum pellucidum and optic nerve abnormalities, though without including the hypothalamic-pituitary dysfunction that completes the modern triad [2]. De Morsier syndrome, or SOD, is a rare congenital midline malformation with an estimated incidence of approximately 1 in 10,000 live births [3]. Classically defined by a triad comprising optic nerve hypoplasia, structural brain abnormalities (notably agenesis of the septum pellucidum), and various endocrine deficits resulting from hypothalamic-pituitary axis involvement, its phenotypic spectrum remains extremely heterogeneous [4]. Although visual disturbances and growth retardation are the most common presenting signs, the clinical presentation during the neonatal period can be misleading. Among these atypical manifestations, neonatal cholestasis poses a major diagnostic challenge [5]. Often associated with central hypothyroidism or corticotropic insufficiency, this functional hepatic impairment can mimic obstructive biliary disease, thereby delaying appropriate management [6].

## Case presentation

A two-month-old male infant was referred to the pediatric department for the evaluation of persistent neonatal cholestatic jaundice, associated with progressive weight faltering and recurrent fasting hypoglycemia. The patient was born at full term (37 weeks of gestation) via an uncomplicated vaginal delivery. His birth weight was appropriate for gestational age at 2,800 g. There was no history of parental consanguinity or any family history of endocrine, metabolic, or hepatobiliary disorders.

Initial physical examination revealed frank cutaneous and mucosal jaundice, dark urine, and partially acholic (beige) stools. Anthropometric measurements confirmed a breakdown in the weight growth curve. Abdominal examination showed mild hepatomegaly with a liver span of 7 cm and a smooth lower border, without splenomegaly or collateral venous circulation. Neurological assessment revealed moderate axial hypotonia and bilateral horizontal nystagmus, which had appeared at approximately six weeks of age. No facial dysmorphism was noted.

**Figure 1:**
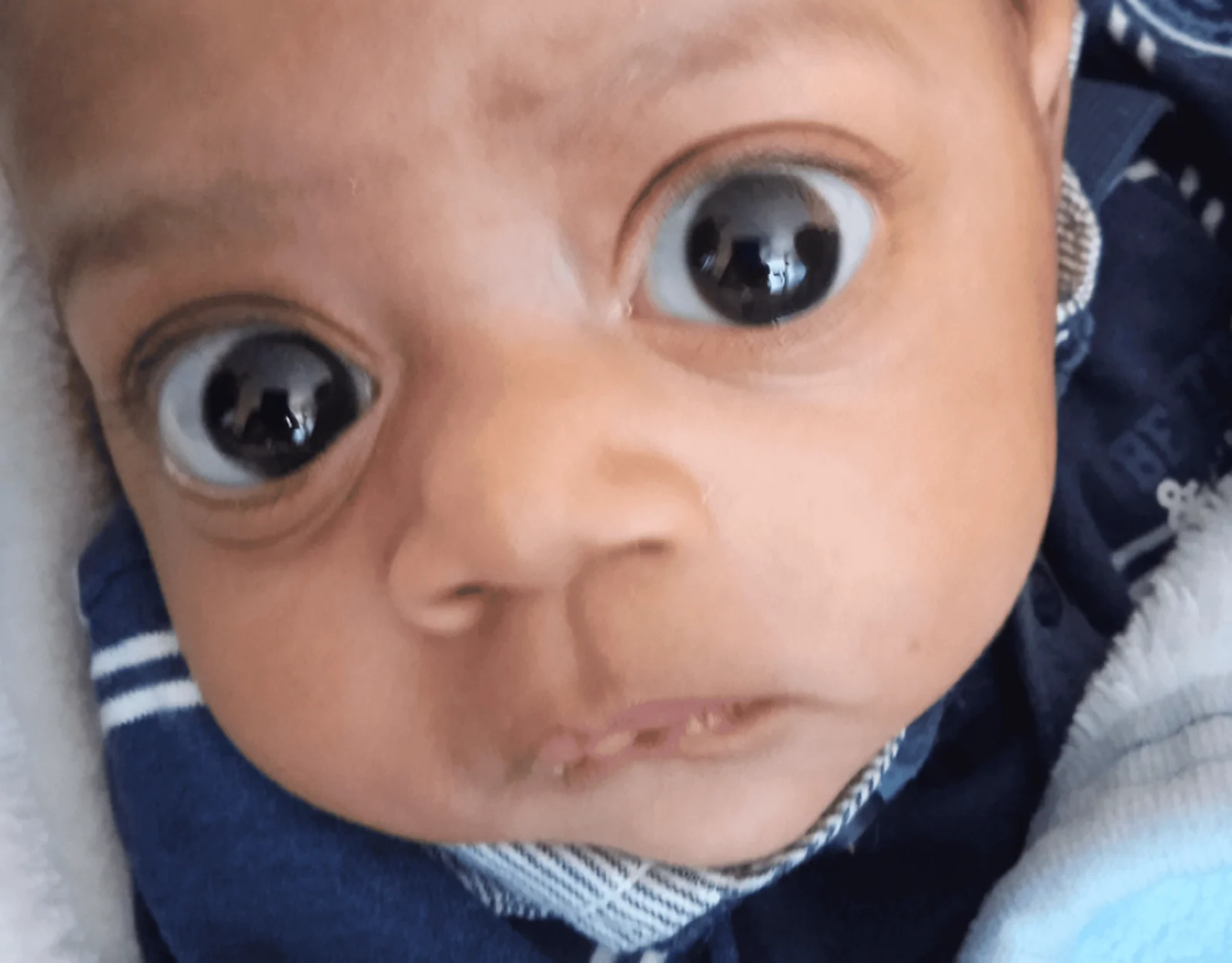
Clinical photograph of the infant's anterior segment. The image demonstrates bilateral megalocornea, characterized by an increased horizontal corneal diameter greater than 13 mm, without signs of corneal clouding, ocular inflammation, or congenital glaucoma. Written informed consent was obtained from the patient's parents for the publication of this case report and any accompanying identifiable images. To ensure proper clinical understanding of congenital megalocornea, the infant's face remained unmasked, as assessing both facial symmetry and precise ocular dimensions is fundamental to recognizing this pathology.

Anterior segment examination using a slit lamp revealed bilateral megalocornea, characterized by a horizontal corneal diameter greater than 13 mm (Figure 1).

Ophthalmic examination confirmed bilateral megalocornea; however, intraocular pressure was normal with no signs of ocular hypertonia, clinically ruling out secondary congenital glaucoma. Consequently, a complementary B-scan ultrasonography was not required during the initial neonatal workup, though long-term ophthalmic surveillance remains indicated.

**Figure 2:**
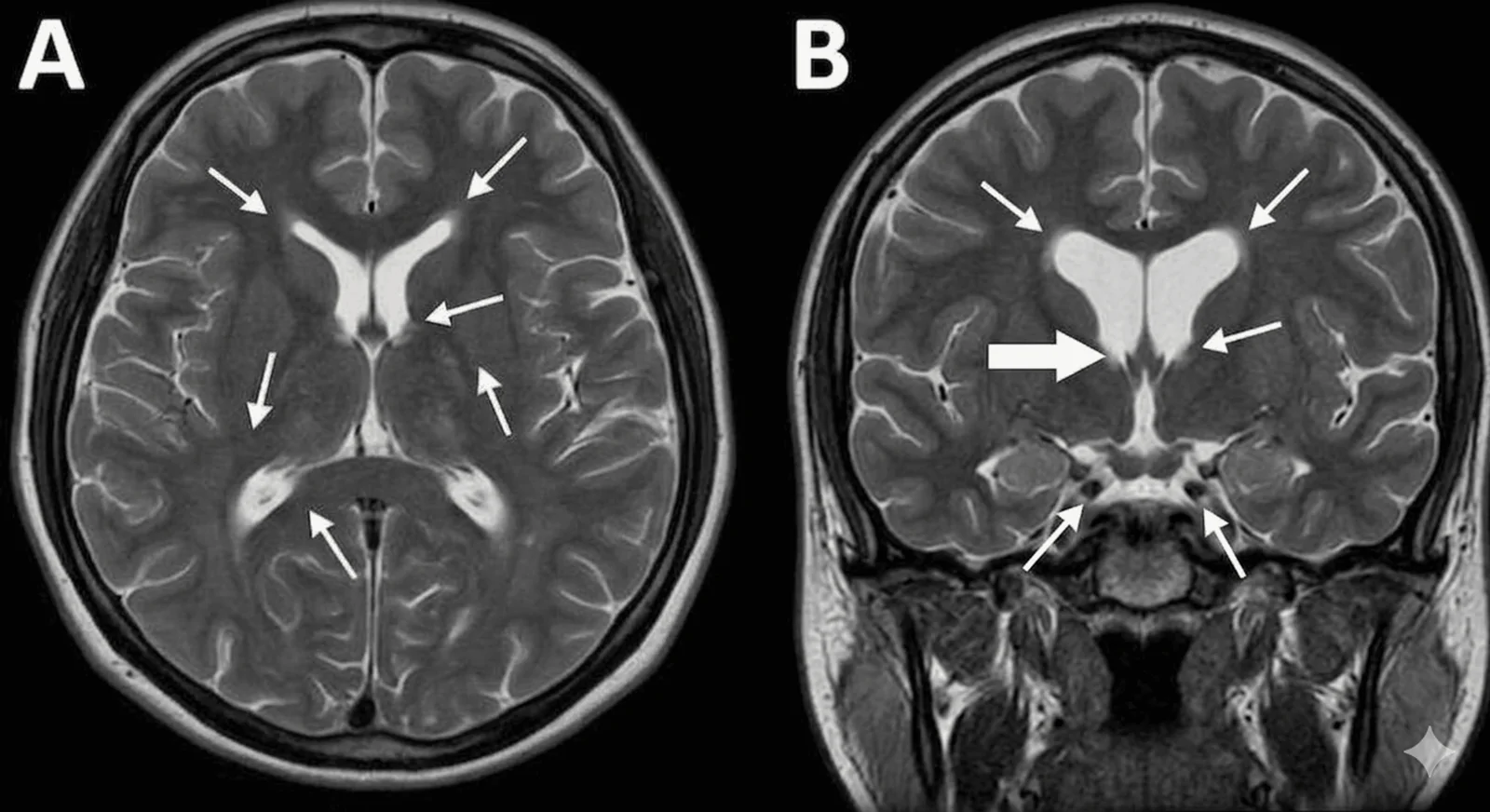
Comprehensive T2-weighted axial MRI analysis. Brain MRI findings confirming septo-optic dysplasia. (A) The axial T2-weighted sequence image shows preserved cortical and deep gray matter structures, highlighting the bilateral prominent ventricles (arrows). (B) A complementary slice demonstrates the characteristic absence of the septum pellucidum and structural midline anomalies, establishing the diagnosis of De Morsier syndrome. The thick white arrow indicates the absence of the septum pellucidum, resulting in characteristic communication and fusion of the lateral ventricles across the midline. The thin white arrows indicate bilateral hypoplasia of the optic nerves (lower arrows) and secondary dysmorphic dilation of the frontal horns of the lateral ventricles (upper arrows).

Biochemical investigations revealed severe neonatal cholestasis with a conjugated bilirubin level of 8.7 µmol/dL (reference range: <0.5 µmol/dL), which remarkably contrasted with a strictly normal GGT of 22 IU/L. Liver synthetic functions, including prothrombin time 72% and albumin levels 35 g/L, were preserved. Given this biological profile and the presence of nystagmus, a first-line endocrine workup was performed. Testing documented central hypothyroidism with a collapsed free thyroxine (FT4) level of 5.2 pmol/L. Growth hormone (GH) deficiency was confirmed using a critical blood sample approach during a severe fasting hypoglycemic episode (plasma glucose: 0.29 g/L (1.6 mmol/L); neonatal reference range: >0.45 g/L (>2.5 mmol/L)). Despite this profound hypoglycemic stimulus, which should physiologically trigger a counter-regulatory GH surge (>20 ng/mL in neonates), the patient's GH level was inappropriately low, establishing a diagnosis of congenital hypopituitarism. This endocrine deficiency was further characterized by concomitant central hypothyroidism, indicated by a low free thyroxine (T4) level of 5.2 pmol/L (reference range: 9.0-19.0 pmol/L) (Table 1). The diagnosis of De Morsier syndrome (SOD) was definitively supported by brain MRI, which revealed characteristic hypothalamic-pituitary midline defects, including the absence of the septum pellucidum (Figure 2).

**Table 1:** Patient's laboratory parameters and reference ranges at admission. *An abnormal value outside the age-appropriate neonatal reference range. GH evaluation showed an actual peak level of 3.5 ng/mL, representing a severely blunted response consistent with congenital GH deficiency.

Lab parameter	Patient value	Reference range	Interpretation
Conjugated bilirubin	8.7 µmol/dL*	<0.5 µmol/dL	High
Free thyroxine (T4)	5.2 pmol/L*	9.0-19.0 pmol/L	Low
Growth hormone (GH)	Low*	Variable	Low
Fasting blood glucose	0.29 g/L* (1.6 mmol/L)	>0.45 g/L (> 2.5 mmol/L)	Severe hypoglycemia
Gamma-glutamyl transferase (GGT)	22 IU/L	<50 IU/L (male), <35 IU/L (female)	Normal
Prothrombin time (PT)	72%	70%-100%	Normal
Albumin	35 g/L	35-50 g/L	Normal (borderline low)

Based on these clinical and biochemical findings, a midline developmental anomaly was highly suspected, and a brain magnetic resonance imaging (MRI) was performed. The MRI confirmed the diagnosis of de Morsier syndrome by revealing complete agenesis of the septum pellucidum, an ectopic posterior pituitary gland, and severe bilateral optic nerve hypoplasia (Figure 2).

**Figure 3:**
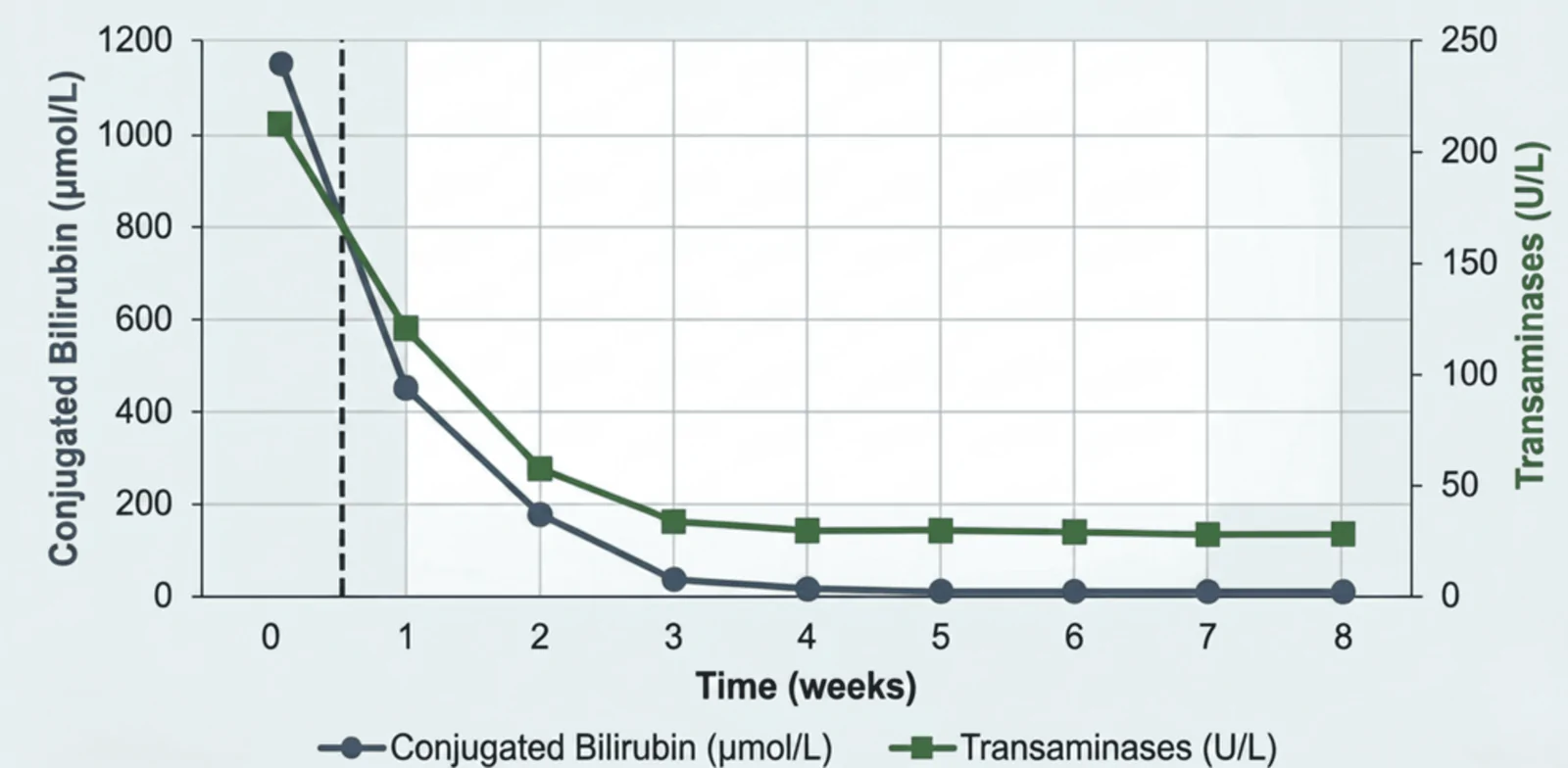
Kinetic evolution of liver function tests before and after targeted hormone replacement therapy. Graph showing the rapid regression of neonatal cholestasis and normalization of total and conjugated bilirubin levels within three weeks following the initiation of hydrocortisone and L-thyroxine replacement therapy. GGT levels remained stable and within normal reference ranges throughout the follow-up period. This figure was created manually by the authors using BioRender software (without generative AI assistance). GGT, gamma-glutamyl transferase; μmol/L, micromoles per liter; U/L, units per liter

A multidisciplinary therapeutic approach was immediately initiated, based on targeted hormone replacement therapy: L-thyroxine at a dose of 10 μg/kg/day. To address the severe recurrent hypoglycemia and the systemic impact of neonatal cholestasis, hydrocortisone was initiated at a stress dose of 5 mg/kg/day. This supra-physiological dosing approach was selected to provide adequate glucocorticoid coverage during acute metabolic stress before transitioning to a standard maintenance regimen. Following the initiation of hormone replacement therapy with hydrocortisone and levothyroxine, serial liver function tests demonstrated progressive and rapid improvement. Full biochemical normalization of conjugated bilirubin, serum transaminases, and liver enzymes, alongside complete clinical resolution of neonatal cholestasis, was achieved exactly three weeks after treatment initiation, along with stabilization of blood glucose levels and resumption of weight gain (Figure 3).

## Discussion

The age at diagnosis for our patient (two months) falls within the early window for forms revealed by endocrine-hepatic involvement. The literature highlights significant temporal variability depending on the dominant presenting sign. In a retrospective cohort reported by Nalawade and Bhate, the median age at SOD diagnosis was five years when isolated visual impairment or growth failure constituted the primary reason for consultation [7]. Conversely, Al-Senawi et al. demonstrated that a clinical presentation combining neonatal hypoglycemia and prolonged jaundice allows for a much earlier diagnosis, typically between three months and one year of age, mirroring the timeline observed in our case [8]. The diagnosis should be clinically suspected in a newborn presenting with hypoglycemia, jaundice, microcephaly, undescended testes, or nystagmus-whether or not associated with a midline defect such as a cleft lip or palate. In such clinical circumstances, an endocrine workup and an ophthalmological consultation are indicated [9].

The overall phenotypic expression of SOD is highly heterogeneous. Epidemiological data from the EUROCAT study by Garne et al. focus on the prevalence and European distribution of the anomaly [10]. However, clinical data from registries such as Orphanet indicate that the complete triad (visual, pituitary, and midline brain defects) is fully present in only 30% of SOD cases [11]. Isolated or combined endocrine deficiencies affect approximately 50% to 55% of patients, predominantly featuring GH deficiency and adrenocorticotropic hormone (ACTH) insufficiency [12].

The primary originality of our observation lies in its association with bilateral megalocornea without hypertonia, characterized by a horizontal corneal diameter greater than 13 mm. In SOD, the classic ophthalmological involvement is limited to optic nerve hypoplasia, which is present in 85% to 90% of cases [13]. The association with structural anomalies of the anterior segment of the eye, such as megalocornea, remains an exceptional manifestation. It reflects a very early developmental insult occurring between the fourth and eighth weeks of gestation [14]. Although more complex ocular malformations or congenital glaucoma are occasionally described, finding an isolated and clear megalocornea during SOD necessitates strict ophthalmological follow-up to rule out a delayed onset of ocular hypertonia [15].

Under targeted hormone replacement therapy with hydrocortisone and L-thyroxine, a spectacular regression of cholestasis was achieved within three weeks in our infant. This resolution timeframe is consistent with data from Ellaway et al., who reported a complete normalization of liver function tests and the clearance of jaundice within an average interval of two to six weeks after initiating glucocorticoid replacement [16]. This rapid reversibility confirms the purely functional and non-obstructive nature of the hepatic involvement [17].

## Conclusions

Neonatal cholestasis with normal GGT levels represents a critical diagnostic warning sign that should immediately evoke congenital hypopituitarism and midline malformations, such as de Morsier syndrome (SOD). Clinical vigilance is crucial during the neonatal period, as early recognition allows for timely, targeted hormone replacement therapy. This intervention is essential to ensure the rapid and complete reversibility of functional hepatic impairment, avoiding unnecessary invasive investigations while significantly improving the long-term neurodevelopmental outcome of these patients. Furthermore, atypical phenotypic associations, including structural anomalies of the anterior segment of the eye, such as megalocornea, underscore the clinical heterogeneity of this syndrome and emphasize the absolute necessity of a stringent, lifelong multidisciplinary ophthalmological and endocrine follow-up.
